# Herbert Melhuish Golding

**Published:** 1971-01

**Authors:** 


### Obituary

### HERBERT MELHUISH GOLDING

Herbert Melhuish Golding was born on 6th April, 1897, and his passing took place on 7th April, 1970, at the age of 73. In the first world war, after serving with the Glo'sters, he was seconded to the Royal Flying Corps, where he was awarded the Distinguished Flying Cross, and promoted to the rank of Flight Commander, for conspicuous gallantry and devotion to duty.

After the war he studied medicine at the University of Bristol, and, as well as obtaining the conjoint diploma, he graduated M.B., Ch.B. in 1924. Then for a brief period he served as Missionary Physician in China. He was very happy in his work among the Chinese people whom he liked and admired greatly, and he might well have devoted his life to this service, but it was not to be. Suddenly he was afflicted with severe illnes which necessitated his relinquishing his work and returning to England. Fortunately, in due course, he made a complete recovery, and then went into general practice in Bristol, where he served till his retirement three years ago.

Space does not permit the recording here of all the positions which he held, nor of all his much merited honours, but just a few of them should be mentioned as they exemplify his devoted service. He was elected chairman of the Bristol Division of the B.M.A. in 1941, and president of the Bristol, Bath and Somerset Branch in 1951. As anyone who knew him would expect, he was particularly interested in the work of the Charities Committee, and in the same year became its chairman. For many years too, he was an active member of the B.M.A. Council, and he was the first Bristol doctor to be made an honorary fellow of the Association. Finally he was county surgeon to The St. John Ambulance Brigade, and, eventually, the honour of Commander of the Order of St. John of Jerusalem was conferred upon him.

One might be tempted to wonder how he could embrace all these duties together with those of a busy practitioner of medicine, yet he succeeded admirably because of his vision, imagination, and above all the selfless devotion to the ideals for which he lived. One recalls that, wherever one met him in other days, he always wore the attractive tie of the R.F.C. with its field of pale blue, cut by the thin red line, and bordered by the dark blue on either side, and, when it was mentioned, he always said, "I'm very proud of it", as indeed he had every reason to be. He was a most able and devoted doctor, and it was both a privilege and an honour to meet him in the world of practice. In his unobtrusive way he conveyed, with his quiet charm of manner, such sincerity and kindness, that for long afterwards the benign glow of his presence was still a beacon light that illumined all the mists of the mundane world. One knew that if he had spent all his days in a far off mission station he would have been just as dedicated, for that was the expression of himself in his life's work. He was devoted to his wife and his three sons, two of whom are of his calling, and to them all we extend our deep and sincere sympathy for the loss of one so near and dear, who wrought always with Eternity in his Heart.

